# Molecular characterization of the *Pratylenchus vulnus* populations on cereals in Turkey

**DOI:** 10.21307/jofnem-2020-084

**Published:** 2020-08-25

**Authors:** Mehmet Sait Karaca, Elif Yavuzaslanoglu, Gul Imriz, Ozlem Ates Sonmezoglu

**Affiliations:** 1 Department of Plant Protection, Ministry of Agriculture and Forestry, International Bahri Dagdas Agricultural Research Institute, Konya, Turkey; 2 Department of Plant and Animal Production, Vocational School, Karamanoglu Mehmetbey University, Karaman, Turkey; 3 Department of Plant Protection, Dicle University, Faculty of Agriculture, Diyarbakir, Turkey; 4 Department of Bioengineering, Faculty of Engineering, Karamanoglu Mehmetbey University, Karaman, Turkey

**Keywords:** Barley, D2-D3 expansion region of 28S rRNA, Detection, Real-time PCR, Sequencing, Walnut root lesion nematode, Wheat

## Abstract

*Pratylenchus vulnus* (walnut root lesion nematode) is one of the most damaging root lesion nematode species worldwide. In this study, 17 populations of *P. vulnus* obtained from wheat and barley cultivated fields in 2016 to 2017 in Turkey (Karaman and Konya provinces) were identified using real-time PCR and melting curve analysis. Samples provided a single peak at 87.3˚C with real-time PCR. D2 to D3 expansion segments of the 28S rRNA of one population from Cihanbeyli district in Konya province was sequenced and recorded in GenBank (Accession number: MT320536.1). Alignments of the population was identical 98.66% to the populations of *P. vulnus* available in GenBank (Accs. nos: LT985479.1 and LT965052.1) and 98.65% (Accs. nos: KY424305.1 and KY424304.1).

Root lesion nematodes of the genus *Pratylenchus* (Filipjev, 1936) are migratory endoparasitic nematodes and the third most damaging nematode species in the world after root knot and cyst nematodes (Castillo and Vovlas, 2007). Currently, according to Janssen et al. (2017), there are 101 species of root lesion nematodes reported worldwide. The most economical important species are *P. penetrans*, *P. thornei*, *P. neglectus*, *P. zeae*, *P. coffeae*, and *P. vulnus* (Jones et al., 2013).

*Pratylenchus vulnus* infects the cortex of the plant roots and causes necrosis of roots, reduced root system, yellowed leaves, whole plant dwarfing, and plant dead (CABI, 2019). It reproduces on a wide range of plant species from strawberry to walnut on many field crops and forest trees (Pinochet et al., 1992).

The genus *Pratylenchus* spp. has high intra specific variation. Additionally, low number of diagnostic features depending on the reproductive strategy of the species requires molecular identification (Castillo and Vovlas, 2007). Molecular techniques as RAPD-PCR and sequencing of D2 to D3 expansion segments of the 28S rRNA was used for the identification of *P. vulnus* on different plant species (Subbotin et al., 2008; Bakooie et al., 2012; Lopez-Nicora et al., 2012). Moreover, real-time PCR provides sensitive identification of the species with species-specific primers using 1/128 of the DNA of one nematode (Huang and Yan, 2017).

*Pratylenchus vulnus* (Allen and Jensen, 1951) (walnut root lesion nematode) has been reported in China, India, Iran, Israel, Japan, Korea, Kyrgyzstan, Pakistan, Sri Lanka, Cameron, Egypt, Kenya, Reunion, Canada, USA, Cuba, Argentina, Brazil, Uruguay, Belgium, Bulgaria, Denmark, Finland, Russia, France, Germany, Greece, Italy, the Netherlands, Norway, Poland, Slovenia, Spain, UK, Australia, and New Zealand (CABI, 2019).

In Turkey, *P. vulnus* has been reported infected pepper (*Capsicum annum*) and rose (*Rosa* sp.) in Istanbul province, ornamental plants in Izmir province, olive in Samsun and sesame (*Sesamum indicum*) in Antalya and Mersin provinces (Saltukoglu, 1974; Borazanci, 1977; Kepenekci, 2001, 2002). It is firstly recorded on cereals in Turkey by Yavuzaslanoglu et al. (2020) using species-specific PCR technique. However, positive control specimen was not available in the study. Real-time PCR with positive control was performed for the 17 *P. vulnus* populations in the current study. In addition, one population was sequenced and recorded in GenBank.

## Material and methods

### Nematode populations

A total of 17 nematode populations obtained from wheat and barley cultivated fields in Konya and Karaman provinces in Central Anatolian Plateau in Turkey on April in 2016 and 2017 were investigated. Two populations were from Center (lat: 37.256700, lon: 33.402159, barley soil) and Ayrancı (lat: 37. 461495, lon: 33. 899667, barley soil) districts in Karaman province and 15 populations were from Çumra (lat: 37. 652138, lon: 32. 810197, wheat soil), Güneysınır (lat: 37. 267285, lon: 32. 703158, wheat plant), Bozkır (lat: 37.215825, lon: 32. 566730, wheat plant), Yalıhöyük (lat: 37.334460, lon: 32.094510, wheat plant), Beyşehir (lat: 37. 708799, lon: 31. 711387, barley plant), Yunak (lat: 38.813822, lon: 31.753218, wheat plant), Kulu (lat: 39.083393, lon: 32.985203, wheat plant; lat: 39.065963, lon: 33.054807, wheat plant; lat: 39.039809, lon: 33.040935, wheat soil; lat: 38.965648, lon: 33.008739, barley plant), Cihanbeyli (lat: 38.627013, lon: 32.920812, wheat plant; lat: 38.468969, lon: 32.833310, wheat plant), Karatay (lat: 37.877460, lon: 32.914944, wheat plant; lat: 37.978114, lon: 32.710529, barley plant), and Kadınhanı (lat: 38.573150, lon: 32.276917, wheat plant) districts in Konya province.

Nematodes were previously identified with PCR fragments at 287 bp using species-specific D3b-R/Pvul-F primer for *P. vulnus* (Yavuzaslanoglu et al., 2020).

### DNA extraction

Total genomic DNA was extracted from five individual nematodes in 30 µl extraction buffer as described by Yavuzaslanoglu et al. (2018). The sample was placed at −20°C for 1 hr and then incubated at 65°C for 1 hr. The proteinase was deactivated at 95°C for 10 min. DNA template was re-suspended in 20 µl TE (10 mM Tris-HCl, 1 mM EDTA, pH: 8.0) (Al-Banna et al., 1997). Prepared DNA suspension was preserved at −20°C until use.

### Real-time PCR

Real-time PCR experiment was set up with DNAs of 17 nematode populations. Negative amplification control (NAC) included distilled water and positive amplification control (PAC) included *P. vulnus* DNA. Samples were processed using Roche 480 real-time PCR. Study was carried out using Clear Detections nematode species-specific real-time PCR diagnostic kit (Product code: RT-N-D-2006, Wageningen, The Netherlands).

The PCR mixture including the nematode specific primer set was vortexed for 2 sec and transferred 15 µL into each well. A 5 µL of each DNA sample were added into their designated well. Real-Time PCR was run including the following steps; initial template denaturation for 3 min at 95°C, 35 cycles of amplification were DNA denaturation for 10 sec at 95°C, annealing for 60 sec at 63°C, and extension for 30 sec at 72°C. DNA melting curve analysis of the amplicon was performed by increasing the temperature from 72 to 95°C at 0.2 to 0.5°C/sec (ramp). The fluorescent signal was measured using the FAM or SYBR/FAM channel, after every cycle and after every temperature increment of the PCR melting curve.

### Sequence alignment

Sequence alignments of one of the *P. vulnus* population from Cihanbeyli district in Konya province (sample number: 184) were determined.

Nematode DNAs was purified using ExoSAP-ITTM PCR Product Cleanup Reagent (Thermo Fisher Scientific, USA) prior to sequence alignment.

Purified DNA samples were sequenced using ABI3730XL Sanger sequencing device and BigDye Terminator v3.1 Cycle sequencing kit (Applied Biosystems, Foster City, CA) in Macrogen laboratory in the Netherlands.

The sequences were deposited into the GenBank database and compared with those of the other *P. vulnus* populations available at the GenBank sequence database using the BLAST homology search tool (https://blast.ncbi.nlm.nih.gov/Blast.cgi).

## Results

**Figure 1:**
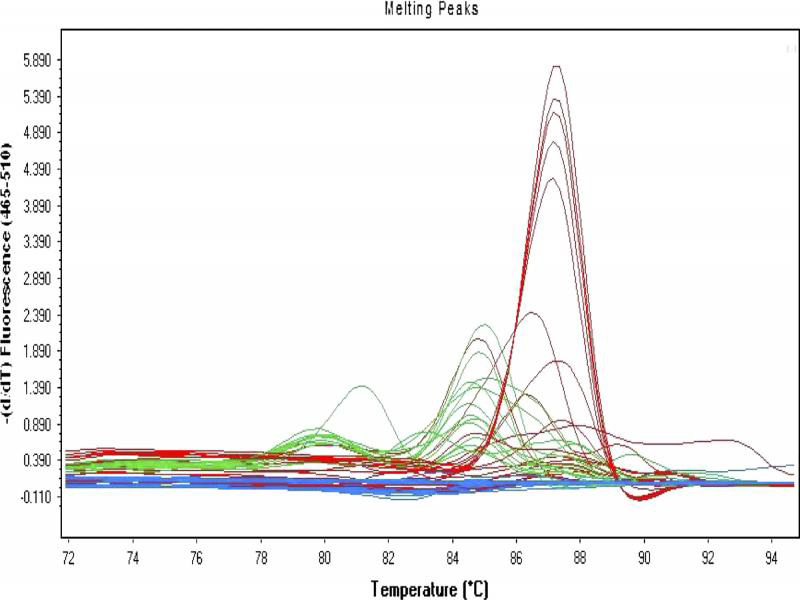
Melting curve of specific amplicons for *Pratylenchus vulnus* with melting temperature at 87.3°C. (green bands indicate positive control, blue bands indicate negative control, and red bands indicate nematode samples).

The 17 *P. vulnus* populations provided single melting peak at 87.3°C indicating that a single amplicon was detected from real-time PCR (Fig. 1).

The sequences of D2 to D3 expansion region of 28S rDNA for the one population of *P. vulnus* (sample no: 184) from Cihanbeyli district of Konya province was examined and deposited in the GenBank database with the accession number of MT320536.1 (https://www.ncbi.nlm.nih.gov/nuccore/1829742534?log$=activity).

Data blast revealed a sequence similarity of 98.66% with *P. vulnus* samples deposited in GenBank (e.g., Accession Nos: LT985479.1 and LT965052.1) and 98.65% (KY424305.1 and KY424304.1).

## Discussion

The real-time PCR was used to estimate the accuracy and sensitivity of molecular identification of *P. vulnus* and provided functional comparison. Qiu et al. (2007) showed specific detection of *P. vulnus* obtained from California orchards using real-time PCR assay and species-specific primers designed from ITS sequences of rDNA. Yan et al. (2012, 2013) and Huang and Yan (2017) reported melting curve analysis for *P. thornei*, *P. neglectus*, and *P. scribneri*, similarly to our observations, single peaks were produced at 88.4, 83.8, and 81.5°C temperatures, respectively.

In many research works, D3 expansion region of 26S rDNA has been using for evaluation and identification among species and genera of nematodes (Huang and Yan, 2017) as well as 28S rRNA (Subbotin et al., 2008; Lopez-Nicora et al., 2012; Janssen et al., 2017).

High similarity rate between the sequence alignments of *P. vulnus* populations from the current study and other geographical areas of the world reported on D2 to D3 expansion regions of 28S rDNA (Al-Banna et al., 1997; Subbotin et al., 2008; Lopez-Nicora et al., 2012; Chihani-Hammas et al., 2018) confirmed the specificity of the molecular identifications.

Molecular identification is specific and reliable in distinguishing nematode species (Nguyen et al., 2017). Nematode species difficult to distinguish morphologically from other species can be successfully identified using molecular characterization.

Detection of *P. vulnus* on wheat and barley as economically important commodities in Turkey is valuable in order to organize future studies on damage potential and control measurements.
